# Exploring the influence of age and diet on gut microbiota development in children during the first 5 years: a study from Yaoundé, Cameroon

**DOI:** 10.3389/fmicb.2024.1512111

**Published:** 2024-12-18

**Authors:** Jorelle Jeanne B. Adjele, Priti Devi, Pallawi Kumari, Aanchal Yadav, Alex D. Tchuenchieu Kamgain, Hippolyte T. Mouafo, Gabriel N. Medoua, Justin J. N. Essia, Nar Singh Chauhan, Rajesh Pandey

**Affiliations:** ^1^Department of Microbiology, Faculty of Science, University of Yaoundé, Yaoundé, Cameroon; ^2^Centre for Food, Food Security, and Nutrition Research, Institute of Medical Research and Medicinal Plant Studies (IMPM), Yaoundé, Cameroon; ^3^Division of Immunology and Infectious Disease Biology, INtegrative GENomics of HOst-PathogEn (INGEN-HOPE) Laboratory, CSIR-Institute of Genomics and Integrative Biology (CSIR-IGIB), New Delhi, India; ^4^Academy of Scientific and Innovative Research (AcSIR), Ghaziabad, India; ^5^Indraprastha Institute of Information Technology Delhi, New Delhi, India; ^6^Food Evolution Research Laboratory (FERL), University of Johannesburg, Johannesburg, South Africa; ^7^Department of Biochemistry, Maharshi Dayanand University, Rohtak, India

**Keywords:** gut microbiota, 16S rDNA gene sequencing, age, diet, children

## Abstract

**Introduction:**

The development of the human gut microbiota is shaped by factors like delivery mode, infant feeding practices, maternal diet, and environmental conditions. Diet plays a pivotal role in determining the diversity and composition of the gut microbiome, which in turn impacts immune development and overall health during this critical period. The early years, which are vital for microbial shaping, highlight a gap in understanding how the shift from milk-based diets to solid foods influences gut microbiota development in infants and young children, particularly in Yaoundé, Cameroon.

**Methods:**

This study involved an analysis of the gut microbiota composition in 70 children aged ≤5 years through 16S rDNA gene metagenomic sequencing of fecal metagenomic DNA. The participants were grouped into four age categories: 0–6 months, 7–12 months, 13–24 months, and 25–60 months.

**Results:**

We observed a reduction in microbial diversity in the younger age groups, which increased progressively with age. At the taxonomic level, our analysis identified *Firmicutes* as the predominant phylum, with its abundance rising in older age groups, suggesting a maturation of the microbiota characterized by distinct genera associations. In the 0–6 month age group, we noted an enrichment of *Lactobacilli* and *Bifidobacteria*, which may play a crucial role in modulating and supporting immune system development during infancy. After 6 months, we found a higher prevalence of *Clostridium*, *Bacillus*, *Roseburia*, and *Faecalibacterium*, which are associated with fiber fermentation and the production of short-chain fatty acids (SCFAs).

**Conclusion:**

These findings underscore the influence of milk products and complementary diets on gut microbiota across various age groups, promoting increased diversity essential for healthy gut development. More such studies in the LMICs would augment and strengthen understanding towards functional microbiome.

## Introduction

The early years of an individual’s life are often known to be a critical window of opportunity for the fundamental development and maturation of a child (Fernandez-Jimenez et al., 2018). During this period, the gut microbiota undergoes significant changes, involving microbial modulation, temporal microorganisms’ acquisition and diversification of microbes (Dogra et al., 2021). Microbes, within the intricate ecosystem, engage in symbiotic relationships, undertaking vital functions such as micronutrient absorption, and vitamin synthesis necessary for proper functioning of the body (Jandhyala et al., 2015).

Particularly, in infants and children under the age of 5 years, these microbes play an important role in stimulating the immune system and significantly influencing their overall development (Derrien et al., 2019). However, this symbiosis can be influenced by a variety of factors, including mode of delivery (vaginal birth vs. cesarean section), breastfeeding vs. formula feeding, antibiotic use, maternal microbiota and health status, and notably, the child’s dietary habits being the major influencer among these variables (Toubon et al., 2023).

Ensuring proper nutrition during the initial years is paramount for the well-being of children under 5 years. According to International Organizations such as World Health Organization (WHO), children should be exclusively breastfed for the first 6 months and continue breastfeeding up to 2 years, supplemented with the appropriate complementary foods (Bhandari, 2016). The introduction of breastmilk exposes the gut microbiota of a newborn to bacteria like *Lactobacilli*, and *Bifidobacteria* species, while formulated milk leads to an enrichment of *Enterobacterium* and *Clostridium* species. The transition from milk-adapted to adult-like gut microbiota during complementary feeding stages (6–24 months) involves increased diversity in the bacterial families like *Lachnospiraceae*, *Ruminococcaceae*, and *Bacteroidaceae* (Laursen, 2021).

In developing countries, child nutrition is often underestimated and depends mainly on the parent finances and education, leading to deviations from the recommended guidelines (Alderman and Headey, 2017). Suboptimal food intake can disrupt intestinal flora, hampering nutrient absorption and vitamin synthesis, contributing to child malnutrition (Iddrisu et al., 2021). In sub-Saharan Africa, specifically in countries like Cameroon, resource-limited environments make infants and young children susceptible to malnutrition, leading to growth challenges between 6 to 24 months (Siddiqui et al., 2020). Parents in these regions often used inexpensive industrially produced foods or resorted to local complementary foods (Oladiran and Emmambux 2020). Although studies like De Fillipo’s show regional diet impacts on gut microbiota, such research is lacking in Cameroon. Thus, a pertinent question which needs attention is, if diversity of infant feeding practices could be the cause of intestinal dysbiosis, which would lead to persistent malnutrition in this region?

In this background, the present study aims to investigate the potential link between infant feeding practices, gut dysbiosis, and persistent malnutrition among children under 5 years of age in Yaoundé, Cameroon. To explore, elucidate and understand this, we performed the 16S rDNA gene sequencing, wherein we have shown age and diet-related changes in the gut microbiota composition.

## Materials and methods

### Study design

A total of 70 children under the age of 5 were selected for participation in this study from 7 district hospitals of Yaoundé, Cameroon during a period of 5 months, from April to August 2022. Eligible participants had refrained from the use of antibiotics or probiotics for a minimum of 2 months before enrollment, and there were no dietary restrictions imposed on them.

### Ethics statement

This study was reviewed and approved by the Centre Regional Ethics Committee for Human Health Research, Yaoundé Cameroon, Reference number 2243/CRERSHC/2021. In compliance with the Institute Ethics Committee’s norms and regulations, written informed consent was obtained from parents of all the children participating in the study.

### Sample collection and sample processing

The stool samples were collected from participants at their place of residence, who were provided with sterile empty containers. These samples were promptly transported to the laboratory under refrigerated conditions within a 2-h window. Subsequently, the stool specimens were barcoded to ensure blinding and frozen at −80°C.

### DNA isolation

Approximately 200–250 mg of stool sample was fully homogenized with 0.1-mm diameter glass beads. Fecal DNA was subsequently extracted using the QIAamp PowerFecal Pro DNA Kit (Qiagen, Cat. No. 51804) according to the manufacturer instructions. The concentration and quality of the extracted genomic DNA was determined by agarose gel electrophoresis and spectrophotometric analysis (NanoDrop Technologies, Wilmington, DE, United States). All extracted DNA samples were stored at −20°C until use for further experiments.

### 16S metagenomic sequencing

For 16S rDNA sequencing, the DNA was quantified and adjusted to 5 ng/μl using Tris buffer (10 mM with pH 8.5) in a reaction volume of 25 ul containing, 2 × KAPA HiFi HotStart ReadyMix, (Roche, Cat. No. 07958935001; KK2602) and primers. The gene-specific sequences used targeted the V3 and V4 region. The V3–V4 hyper-variable regions of the 16S rDNA gene were amplified 16S Amplicon PCR Forward Primer = 5′-TCGTCGGCAGCGTCAGATGTGTATAAGAGACAGCCTACGGGNGGCWGCAG-3′ Amplicon PCR Reverse Primer = 5′-GTCTCGTGGGCTCGGAGATGTGTATAAGAGACAGGACTACHVGGGTATCTAATC-3′. This variable region was amplified by PCR using Illumina adapter overhang nucleotide sequences, the main reason for using this region was that it contains the maximum nucleotide heterogeneity and displays the maximum discriminatory power for appropriate size. In this process, the thermal cycling consisted of initial denaturation at 95°C for 3 min, followed by 25 cycles of denaturation at 95°C for 30 s, the annealing process at 55°C for 30 s and the extension at 72°C for 30 s with the final extension at 72°C for 5 min. PCR purification was performed using AMPure XP beads (Beckman Coulter, Cat. No. A63881). The concentration of metagenomic sequencing libraries was assessed using the Qubit dsDNA HS Assay Kit on a Qubit 3.0 fluorometer and KAPA HiFi HotStart ReadyMix (Roche, Cat. No. 07958935001; KK2602). DNA libraries were constructed according to the Illumina protocols, and sequencing was conducted by an Illumina MiSeq platform (2 × 300 bp paired end chemistry with multiplexed pooled samples) and 1 μL of the PCR product was checked for the appropriate size with a Bioanalyzer chip (Agilent DNA 1000 kit catalog # 5067-1504).

### Metagenomic analysis

Prior to sequence analysis, raw reads were quality filtered and trimmed using Trimmomatic v0.39 to remove adaptor and low-quality sequences (Bolger et al., 2014). It was then aligned with HISAT2 to remove any plausible human host RNA reads by mapping reads onto the human reference genome GRCh38 (Kim et al., 2015). Samtools was used to filter human unaligned reads for downstream analysis to identify the microbes, microbial mapping and taxonomic classification. Following quality control steps, Kraken2, a taxonomic classifier that maps sequencing k-mers to genomic databases, was used to assign taxonomy on filtered and pre-processed reads (Wood et al., 2019). The database was downloaded which is built from the refseq bacteria, archaea and viral libraries. The Kraken2 function was used to run the filtered reads against this database and assign taxonomy. While Kraken2 does not estimate species abundances, Bracken2 (Bayesian Reestimation of Abundance with KrakEN) uses the taxonomy assigned by Kraken2 to estimate the number of reads per sample that originate from the individual species (Lu et al., 2017). The Kraken2 database was used to create a Bracken-compatible database using the bracken build function, and the Kraken2 report files for each sample were run against the Bracken database using the bracken function for the phylum, genus and the species level information. The kraken-biom function was used to convert the Bracken report files into a biom file to import into R. CSS normalization was used to normalize the read count generated from the Bracken. Taxonomic diversity analysis through Alpha diversity was performed using the phyloseq, vegan, tidyverse, dplyr packages in R (v4.2.0) and analysis through beta diversity was performed using Adonis2 package in R (v4.2.0). Towards this, the biom file was imported into R via the phyloseq object. Alpha diversity (within-sample diversity) indices, including Shannon, Simpson and Chao-1 index, were calculated on the basis of the species profile for each sample. Beta diversity (between-sample diversity) was calculated as bray-curtis index by the phyloseq, vegan and Adonis2 packages and visualized by principal coordinate analysis (PCoA) plot. Data was exported as csv files and formatting, plotting and visualization was performed in ggplot2, ggpubr, python (3.6) using the seaborn library (0.12.2) and Matplotlib (3.1) built on NymPy (1.11.0).

### Statistical analysis

Kruskal–Wallis test was used to determine the abundance of microbiome and differential abundance of bacterial species across different groups. Statistical significance of beta diversity analysis was determined using PERMANOVA. STAMP (Statistical Analysis of Metagenomic Profiles) analyzed the statistical significance of various genera and species of different age groups between 0–6 months and 13–24 months using the White’s non-parametric *t*-test.

## Results

### Study design and augmented gut microbial diversity in infants across different age groups

**Figure 1:**
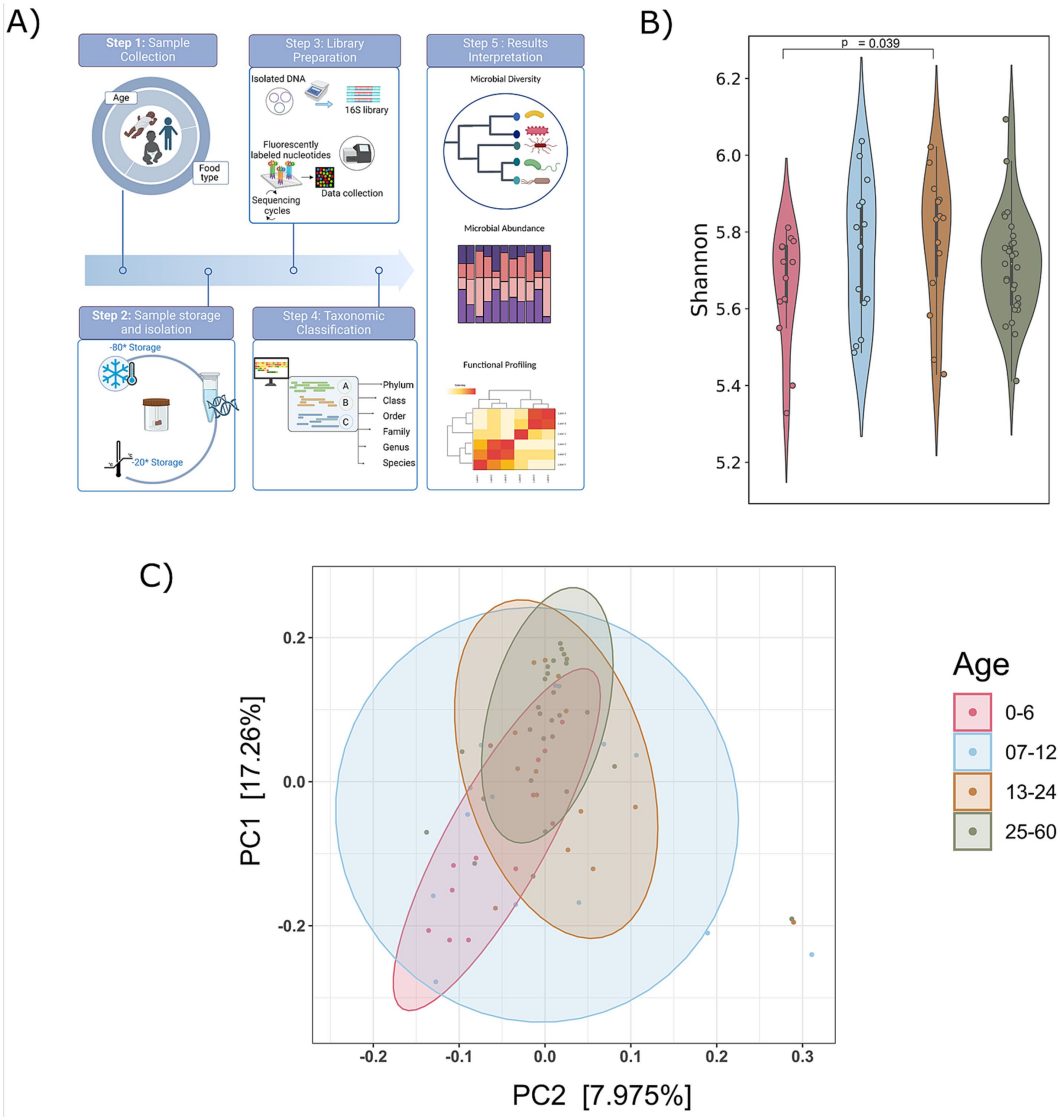
Compendium of the study design and diversity analysis. **(A)** An overview of study design and methods applied. **(B)** Shannon alpha diversity from 0 to 60 months of’ age group was measured (significance, *p* ≤ <0.05; Wilcoxon rank-sum test); and **(C)** principal coordinate analysis plot (Bray–Curtis distances) illustrating the gut microbiome composition across different age groups (0–6, 7–12, 13–24, and 25–60 months). Beta diversity analysis (Bray–Curtis) did not show significant differences (*p* > 0.05).

In our study, we analyzed the gut microbiome by examining fecal samples from 70 children aged 5 years or younger in Yaoundé, Cameroon. A thorough demographic data for the participants regarding age, gender, birth delivery mode, and food type was meticulously collected (Supplementary Table S1). An overview of the study design and methods are illustrated in Figure 1A. Subsequently, 16S rDNA gene sequencing generated ~156,292—reads per sample corresponding to data (Supplementary Table S2).

A rarefaction curve was used to determine if the samples had been sufficiently sequenced to accurately represent their true diversity. The analysis revealed that the libraries in the included samples effectively captured the majority of bacterial diversity, as indicated by the plateau stage reached in almost all the study samples. This affirms that the obtained number of reads is suitable for further analysis (Supplementary Figure S1A).

To analyze the gut microbiome composition in participants aged 5 years or younger, we categorized them into four age groups: infants (0–6, 7–12 months), toddlers (13–24 months), and preschool children (25–60 months). To explore age-related variations in the gut microbiota, alpha diversity (Shannon, Simpson, and Chao indices) and principal coordinates analysis (PCoA) were employed to visualize beta diversity across these age groups (Supplementary Table S3). An increasing trend in alpha diversity was observed with age, with the 13–24 months exhibiting significantly higher alpha diversity compared to the 0–6 age (Figure 1B; Supplementary Figure S1B). However, no significant differences in the beta diversity of gut microbiome communities were observed with the age (Figure 1C). The PCoA for each age group revealed distinct yet overlapping profiles. The alpha diversity exhibits an increasing trend, indicating a rise in both the richness and evenness of microbial composition as individuals age, coupled with the stability of the microbiota.

### Dynamic gut microbiome composition across phylum, genera and species across diverse age groups

**Figure 2:**
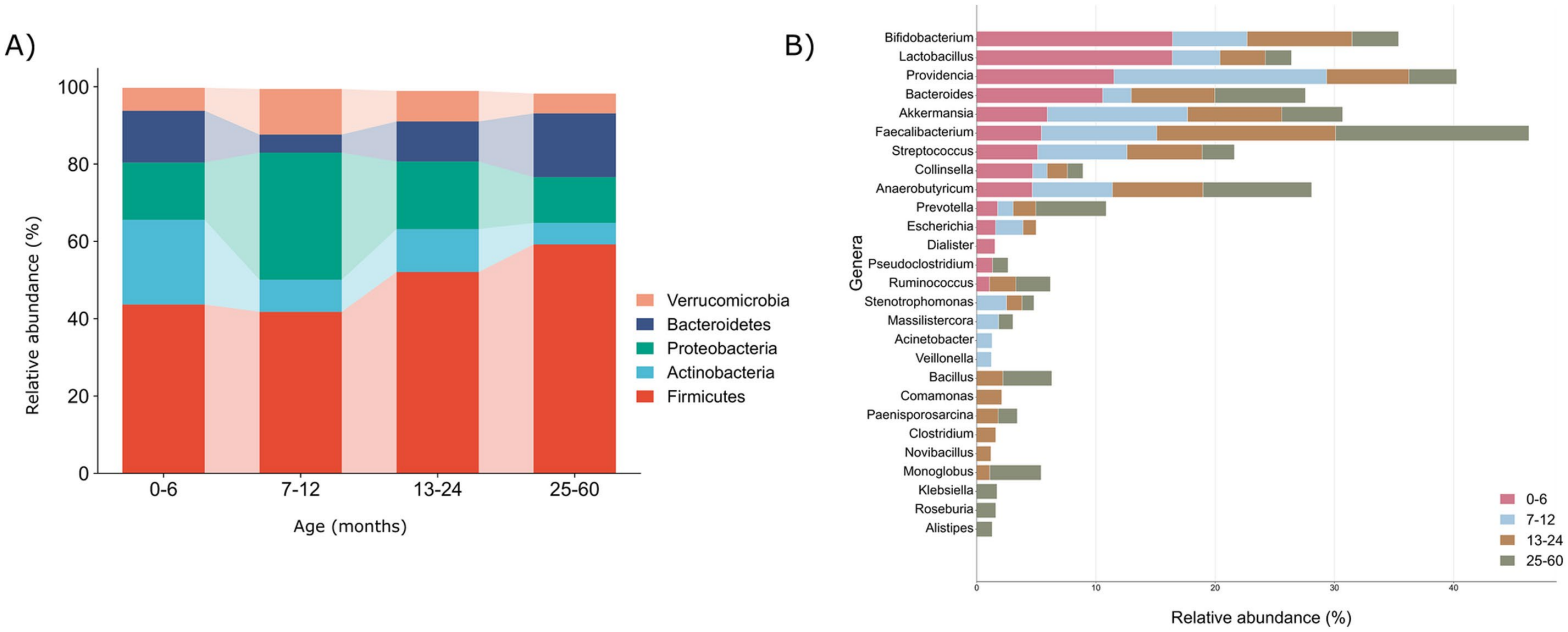
Illustration of microbial profiles at phyla and genera taxonomic levels across various age groups (0–6, 7–12, 13–24, and 25–60) months. **(A)** The relative abundances of the 5 major phyla (>1%) according to age groups. **(B)** The relative abundances of the major genera (>1%) according to the age groups with bold emphasis on the dominant genera in each respective age group.

We carefully examined age-related changes in the gut microbiota composition, focusing on the relative abundance percentages at the phylum and genera levels, aiming to uncover subtle nuances and provide valuable insights into the intricate dynamics across diverse age groups (Supplementary Table S4). Specifically, there was an observed increase in the number of detected phyla with increasing age: 35, 32, 34, and 39 in the 0–6, 7–12, 13–24, and 25–60 month age groups, respectively. This observation suggests a progressive expansion and diversification of gut microbiota phyla over the developmental stages examined (Figures 2A,B and Supplementary Table S4).

Remarkably, our examination revealed consistent presence of five major phyla—Firmicutes (43.67, 41.77, 52.07, 59.21%), Bacteroidetes (13.3, 4.6, 10.3, 16.4%), Actinobacteria (21.9, 8.28, 11.1, 5.57%), Proteobacteria (14.8, 32.8, 17.4%, 11.8), and Verrucomicrobiota (5.9, 11.7, 7.8, 5.09%)—across all the age groups, depicted in the Figure 2A. On top of it, we depicted the percentage relative abundance of phyla per sample to observe deviations across all age groups (Supplementary Figure S2). A noteworthy observation is the consistent prevalence of Firmicutes, surpassing 40% in all the groups, indicating a direct correlation with age. Conversely, Actinobacteria and Proteobacteria demonstrated an inverse relationship with age, barring a substantial increase in Proteobacteria observed in the 7–12 month-old infants. Moreover, Verrucomicrobiota while maintaining an overall range of 5–7%, exhibited a higher relative abundance in the 7–12 month old infants. The observed differences in the phylum-level microbial composition among various age groups prompted a more in-depth exploration at the genus level to elucidate the intricacies of gut microbiota dynamics.

Genus level analysis revealed the cumulative count of 581, 658, 657, and 742 genera within the 0–6, 7–12, 13–24, and 25–60 month, respectively, highlighting a consistent upward trajectory in the genus diversity and abundance with advancing age of the infants. We explored dominant genera with a relative abundance >1%, revealing 14, 15, 19, and 19 genera in the age groups 0–6, 7–12, 13–24, and 25–60, respectively (Figure 2B) and depicted these genera per sample in each group as well (Supplementary Figure S3). Remarkably, 10 anaerobic genera, namely *Bifidobacteria*, *Lactobacilli*, *Providencia*, *Bacteroides*, *Akkermansia*, *Faecalibacterium*, *Streptococcus*, *Collinsella*, *Anaerobutyricum*, and *Prevotella*, were consistently prevalent across all the age groups. Together, these genera collectively represented more than 50% of all the microbial genera in each group, emphasizing their significant contribution and establishing a core microbiota consistently present across children of different ages.

Further, to scrutinize the variation in the abundance of these core genera across distinct age groups, a comparative analysis was conducted by assessing their respective abundance levels. In infants ≤6 months, *Lactobacilli* (16.4%), *Bifidobacteria* (16.4%), and *Bacteroides* (10.5%) emerged as prominent genera. Subsequently, in infants aged 7–12 months, *Providencia* (17.8%) and *Akkermansi*a (11.7%) exhibited initial prevalence. Over the subsequent year, a stabilization of *Faecalibacterium* (14.9% vs. 16.1%), *Anaerobutyricum* (7.5% vs. 9.0%), *Ruminococcus* (2.2% vs. 2.8%), and *Bacillus* (2.1% vs. 4.1%) was observed in the 13–24 and 25–60 month age groups, respectively. Noteworthy, prominent genera like *Lactobacilli* and *Bifidobacteria* during infancy, *Clostridium* (1.5%) notably in the 13–24 month-old toddlers, and *Klebsiella* (1.6%) alongside *Prevotella* (5.8%) in the 25–60 month age group were identified and emphasized in Figure 2B. This highlights a transition toward an adult-like microbiota composition as infants’ progress from infancy to toddlerhood and preschool age.

As children aged beyond 6 months, new genera proliferated, and distinct trends in prevalence emerged across the age groups. Notably, several genera emerged in higher abundance (>1%) after 6 months, including *Stenotrophomonas*, *Massilistercora*, *Acinetobacter*, *Veillonella*, *Bacillus*, *Comamonas*, *Paenisporosarcina*, *Clostridium*, *Novibacillus*, *Monoglobus*, *Klebsiella*, *Alistipes*, and *Roseburia*. In particular, *Acinetobacter* and *Veillonella* were more abundant in the 7–12 months age group, while *Comamonas*, *Clostridium*, and *Novibacillus* dominated the 13–24 months group. For the 25–60 months group, *Klebsiella*, *Alistipes*, and *Roseburia* were prominent (Figure 2B). This highlights dynamic transitions in gut microbiota composition, revealing distinct patterns across different age groups.

**Figure 3:**
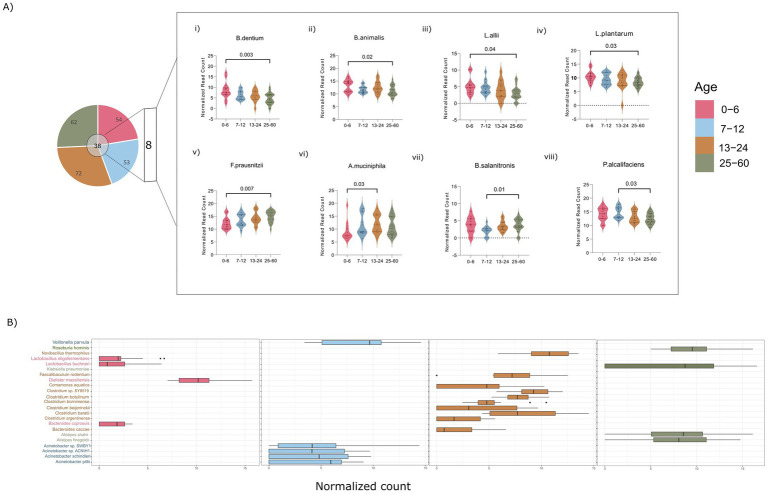
**(A)** Dynamics of the common and unique bacterial species. **(i–v)** Illustrating the significant bacterial species across age groups of 0–6, and 25–60 months **(vi)** across 0–6, and 13–24 months **(vii,viii)** across 7–12, and 25–60 months. **(B)** Box plots showing the distribution of unique species across various age groups of 0–6, 7–12, 13–24, and 25–60 months. (Significance, *p* ≤ 0.05; ANOVA non-parametric test; Kruskal–Wallis test).

To further validate the observed disparities at the species level, a comprehensive examination of species within all genera (>1%) in each group was undertaken. A rigorous filtering criterion was applied, requiring the species to be present in at least 50% of the participants and to have a relative abundance equal to or exceeding 0.1%, thus mitigating potential biases resulting from artifacts. This stringent approach yielded varying numbers of observed species in each age group: 54, 53, 72, and 62 bacterial species in the 0–6, 7–12, 13–24, and 25–60 months old groups, respectively. Notably, there was a substantial difference in the number of species, particularly between the 0–6 and 13–24 month old groups, aligning with the significant alpha diversity observed across these two groups (Figure 3A).

This thorough species-level examination has identified a collective total of 38 common species across all the studied groups. Among these, 8 species demonstrated statistical significance in pairwise comparisons between the different age groups (Figure 3A). Species associated with *Bifidobacteria* (*Bif. dentium* and *Bif. animalis*) and *Lactiplantibacillus* (*Lpb. allii* and *Lpb. plantarum*) demonstrated an inverse relationship with age, exhibiting significantly higher abundance in the 0–6 month old infants compared to those aged 25–60 months (Figure 3A,i–iv). In contrast, *Faecalibacterium prausnitzii* species displayed an age-associated increase, with a significant rise in the 25–60 month-old children compared to those aged 0–6 months, aligning with trends observed at the genus level (Figure 3A,v). *Bacteroides salanitronis*, on the other hand, significantly declined in 7–12 month-old infants and showed a substantial reduction compared to the children aged 25–60 months (Figure 3A,vii). The 13–24 month and 7–12 month age groups was found to be enriched with *Akkermansia muciniphila* and *Providencia alcalifaciens*, respectively, compared to the 0–6 and 25–60 month-old children (Figure 3A,vi,viii). The identification of statistically significant species variations contributes valuable insights into the nuanced shifts occurring in the gut microbiota composition during distinct developmental stages.

Furthermore, a targeted analysis of unique species within each of the aforementioned genera has revealed specific patterns of species abundance in each age groups. Notably, infants aged 0–6 months were found to harbor species within the *Lactobacilli* genus (*Lpl. oligofermentas*, *Lcb. buchneri*), as well as *Bacteroides coprosuis* and *Dialister massiliences* (Figure 3B). In the 7–12 age group, there was an observed enrichment of species belonging to *Veillonella* (*V. parvula*) and *Acinetobacter* (*A. pittii*, *A. schindleri*). Species within the *Clostridium* genus, such as *C. botulinum*, *C. bornimenes*, *C. beijerinckii*, and *Bacteroides* (*Bact. caccae*), were notably associated with the 13–24 month age group. Furthermore, in the 25–60 month age group, *Klebsiella* and *Alistipes* species were prevalent, including *K. pneumoniae*, *A. shahii*, and *A. finegoldii*.

The detailed examination of specific phyla, genera, and individual species highlights nuanced variations in the composition of the gut microbiota across different age ranges. The systematic progression from phylum to species level unveils a discernible trend, providing insights into the enrichment of particular phyla, genera, and species during various stages of development.

### Age-specific changes in the directionality of taxon abundance between the infant (0–6 months) and toddlers (13–24 months)

We observed significant differences in diversity in two specific age ranges (0–6 and 13–24 months), reflecting variations in their microbial compositions. A total of 35 phyla and 581 genera were observed in the 0–6 month-old group, while the 13–24 month-old group exhibited 34 phyla and 657 genera (Supplementary Table S4). Notably, five phyla (>1%)—Firmicutes (43.6% vs. 52.1%), Actinobacteria (21.9% vs. 11.1%), Proteobacteria (11.5% vs. 17.4%), Bacteroidetes (13.3% vs. 10.3%), and Verrucomicrobia (5.9% vs. 7.8%), were found to be prevalent in the 0–6 and 13–24 age groups, respectively.

**Figure 4:**
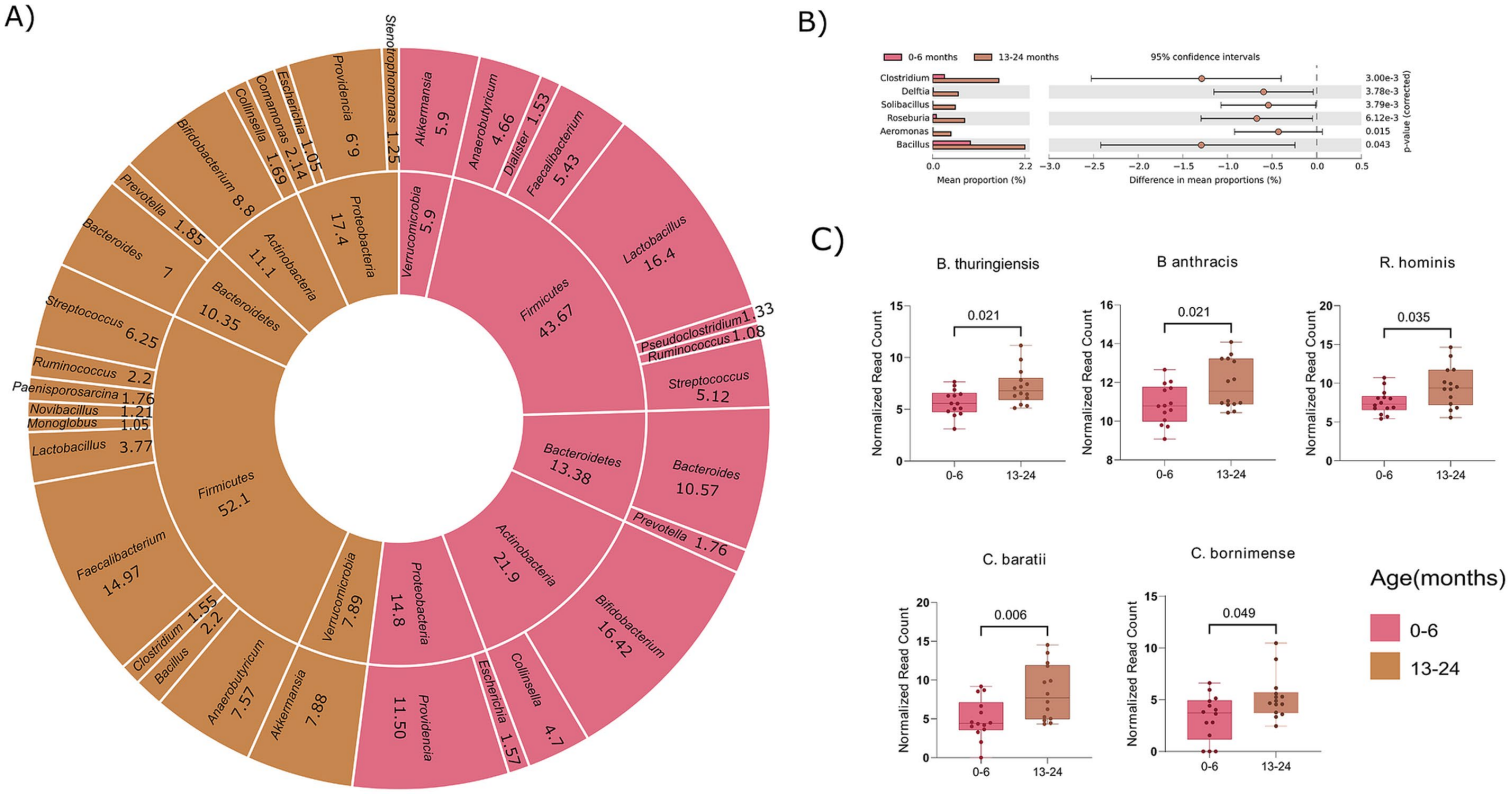
Comparative analysis of microbial composition at phylum, genus, and species levels between age groups 0–6 and 13–24 months. **(A)** Donut plot showing phyla and their corresponding genera (>1%). The inner circle indicates the genera and the outer circle corresponds to the phyla. **(B)** Visualization of significant genera across the two age groups (0–6 and 13–24 months). **(C)** Box plot showing statistical significant bacterial species associated with identified genera. (Significance, *p* ≤ 0.05; non-parametric test; White test).

At the genus level (>1%), variable numbers of genera were observed within the Firmicutes and Proteobacteria phyla in both the age groups. Specifically, the 0–6 month infants had 7 genera in Firmicutes and 2 in Proteobacteria, while the 13–24 months exhibited 10 genera in Firmicutes and 4 in Proteobacteria (Figure 4A). This emphasizes distinct genus-level diversity for these phyla, aligning with the total genera mentioned earlier for their respective age groups. To examine notable differences in abundance among genera, we employed STAMP analysis, which identified 6 genera (*Clostridium*, *Delftia*, *Solibacillus*, *Roseburia*, *Aeromonas*, *Bacillus*) with significant increase in abundance in the 13–24 month-old compared to the 0–6 month-old (Figure 4B). Taking a more in-depth approach, our analysis expanded to the species within these important genera, aiming to identify noteworthy distinctions at the species level. To maintain stringency and uniformity, we filtered out bacterial species corresponding to their genera with a relative cumulative abundance of ≥0.1% and presence in at least 50% of the samples within each group.

In total, 15 species were identified in the 0–6 age group and 18 species in the 13–24 month-old, with all the 15 species from the 0–6 month were also present in the 13–24 age group. Interestingly, 3 unique species namely, *Clostridium argentinense*, *Clostridium beijerinckii*, and *Solibacillus silvestri* were discovered in the 13–24 month-old toddlers compared to the 0–6 month-old infants. Further significance assessment of the 15 common species revealed that 5 species were statistically significant, belonging to *Bacillus* (*B. thuringiensis*, *B. anthracis*), *Clostridium* (*C. baratii*, *C. bornimense*), and *Roseburia* (*R. hominis*) (Figure 4C). This comprehensive analysis reveals specific bacterial species contributing to the differences in microbial composition between the infants (0–6 months) and toddles (13–24 months) groups, attributing significant alpha diversity to variations at the level of genera and species. It will further be interesting to ascertain whether the variance in microbial composition and alpha diversity is attributable solely to age or if diet also contributes to the observed increase/decrease in certain genera from 0–6 months to 13–24 months age groups.

### Influence of diet on gut microbiome dynamics at the genus level

Thus far, our focus on age-related taxonomic distribution among infants, toddlers, and preschool children prompts consideration of the participant’s diet variability, which plays a pivotal role in shaping gut microbiota composition. All participants were exposed to three diets over time: plant-based only, dairy-based only, and plant and animal-based diets. To understand observed patterns, we investigated if they stem solely from age or result from the combined influence of age and diet. This investigation aims to illuminate the interplay and potential synergies between these factors in shaping the gut microbiota composition.

**Figure 5:**
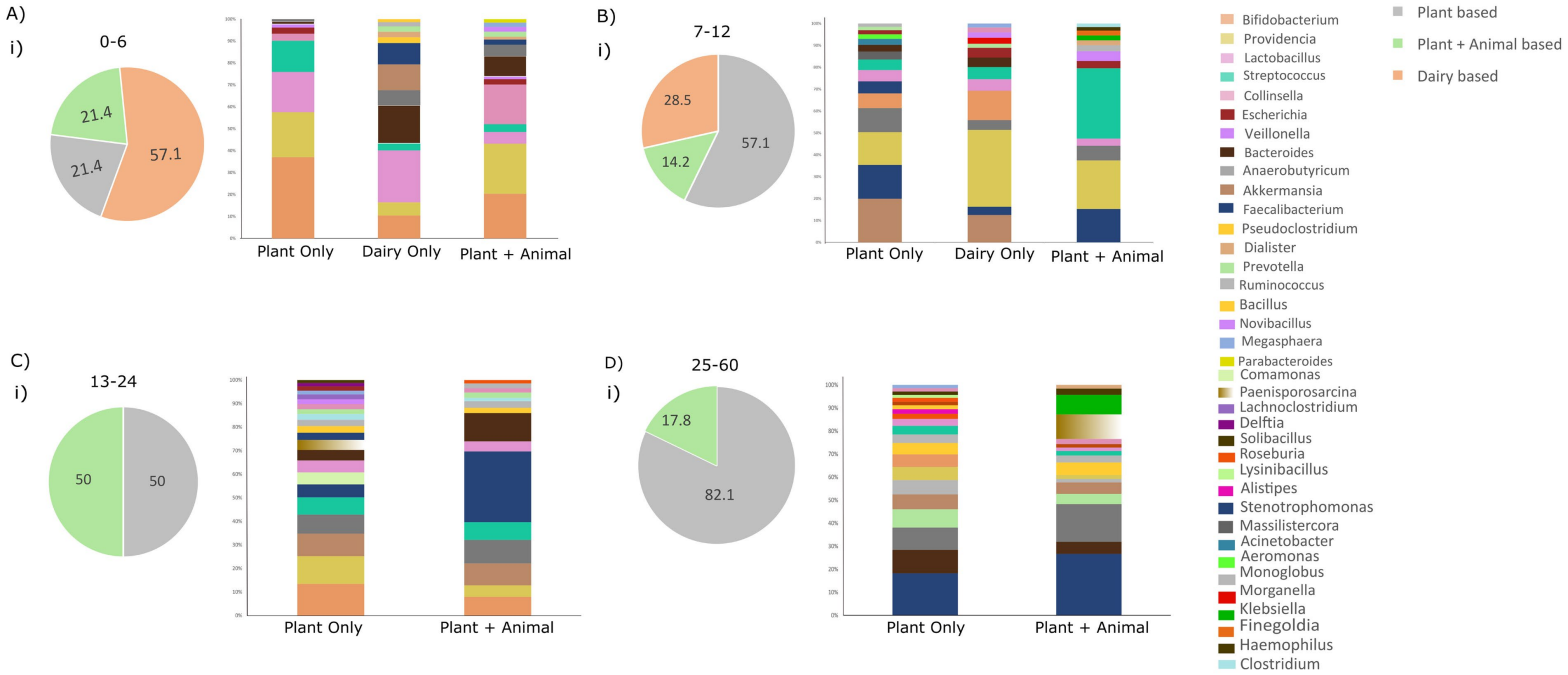
Illustrating dietary patterns (plant-based, dairy-based, and plant + animal-based) across all age groups and the distribution of genera. **(A)** Distribution of diet groups in the age group of 0–6 months and relative abundance of taxonomic genera based on the dietary habits. **(B)** Distribution of diet groups in the age group 7–12 months and relative abundance of taxonomic genera based on the dietary habits. **(C)** Distribution of diet groups in the age group of 7–12 months and relative abundance of taxonomic genera based on the dietary habits. **(D)** Distribution of diet groups in the age group of 25–60 months and relative abundance of taxonomic genera based on the dietary habits.

In this context, it is noteworthy that 57.1% (*n* = 8) of infants aged 0–6 months and 28.5% (*n* = 4) of those aged 7–12 months exclusively followed a dairy-based diet. In contrast, 21.1% (*n* = 3) of infants aged 0–6 months, 57.1% (*n* = 8) of those aged 7–12 months, 50% (*n* = 7) of those aged 13–24 months, and 82.1% (*n* = 23) of those aged 25–60 months adhered exclusively to a plant-based diet. Additionally, 21.4% (*n* = 3) of children aged 0–6 months, 14.2% (*n* = 2) of those aged 7–12 months, 50% (*n* = 7) of those between 13–24 months, and 17.8% (*n* = 5) of age 25–60 months were following both plant and animal-based diets (Figure 5A,i). This analysis provides a comprehensive overview of diet distribution patterns, revealing a predominance of plant-based diets in participants older than 6 months. In pursuit of this, we scrutinized the taxonomic distribution at the phylum and genus levels concerning the three diets within each age group separately (Supplementary Table S5).

Examining the genus level within each age group, distinct prevalent genera emerged as the diet altered, highlighted in Figure 5. In the 0–6 age group, with most infants consuming dairy, prevalent genera were *Lactobacilli* (20.5%), *Bacteroides* (14.9%), *Akkermansia* (10.2%), and *Bifidobacteria* (9.02%), which constitute 50% of total genera. A plant-only diet favored *Bifidobacteria* (33.9%), *Providencia* (18.8%), and *Lactobacilli* (16.8%), while diet with both plants and animals enriched *Providencia* (20.9%), *Bifidobacteria* (18.5%), and *Collinsella* (16.5%), comprising over 50% of genera. This diet variability within the 0–6 age group underscores the influence of diet on shaping the gut microbiota (Figure 5A,i).

Similarly, in the 7–12 age group, a predominantly plant-based diet was observed in most infants, characterized by the prevalence of *Akkermansia* (15.3%), *Faecalibacterium* (11.9%), *Providencia* (11.6%), *Anaerobutyricum* (8.4%), and *Bifidobacteria* (5.2%). Conversely, a dairy-only diet in this age group was enriched with *Providencia* (29.3%), *Bifidobacteria* (11.1%), and *Akkermansia* (10.3%). A small subset of children in this age group had a diet comprising both plant and animal sources, with prevalent genera including *Streptococcus* (28.1%), *Providencia* (19.5%), and *Faecalibacterium* (13.4%) (Figure 5B,i).

In the 13–24 age group, half of the toddlers exclusively followed a plant-based diet, while the other half had a diet comprising both plant and animal sources. In the plant-based diet category, prevalent genera included *Bifidobacteria* (11%), *Providencia* (9.7%), *Akkermansia* (7.8%), *Anaerobutyricum* (6.6%), *Streptococcus* (6.1%), and *Faecalibacterium* (4.5%). In the mixed plant and animal-based diet category, *Faecalibacterium* (25.4%), *Bacteroides* (10.2%), *Anaerobutyricum* (8.4%), and *Akkermansia* (7.8%) were enriched (Figure 5C,i).

For the 25–60 age group, *Faecalibacterium* (14.7%), *Bacteroides* (8.2%), *Anaerobutyricum* (7.9%), *Prevotella* (6.3%), *Akkermansia* (5.2%), *Monoglobus* (5.02%), and *Providencia* (4.5%) were prevalent in the plant-only diet. In contrast, *Faecalibacterium* (22.7%), *Anaerobutyricum* (14.05%), *Paenisporosarcina* (9.0%), and *Klebsiella* (7.2%) were enriched in children with a mixed diet comprising both plant and animal foods. This nuanced analysis illustrates the varied microbial responses to different dietary patterns within specific age groups (Figure 5D,i).

For instance, infants aged 0–6 and 7–12 months commonly consumed dairy products. Yet, examining the gut microbiota composition revealed variations in dominant genera among children exclusively consuming dairy. Similar disparities were noted in other age groups; for the 7–12 month age group, a plant-based diet showed a higher abundance of *Akkermansia*, *Providencia*, and *Faecalibacterium*, distinguishing it from other groups. In the 13–24 month age group, a plant-based diet was associated with the prevalence of *Bifidobacteria*, *Providencia*, *Faecalibacterium*, and *Streptococcus*. In the 25-60-month age group, as children transitioned toward an adult-like microbiota, a predominantly plant-based diet played a significant role in the prevalence of specific genera. This underscores the pivotal role of age in shaping the microbiota, even with a consistent dietary pattern. However, variations in the abundance of prevalent genera under the same diet across different age groups suggest the synergistic effect of age and diet in shaping the gut microbial composition in children below 5 years.

Interestingly, the introduction of animal-based food along with plant-based diets in the 13–24 and 25-60-month-old children highlighted the increased preponderance of specific genera. Notably, *Klebsiella* in the 25–60 month-old children and *Faecalibacterium* in both the 13–24 and 25–60 month age children were found to be more abundant. The noticeable increase in *Faecalibacterium* during the second year of a child’s life (13–24 months), as demonstrated in the preceding result (14.97%), may be influenced by the introduction of animal-based diet among these children, as depicted in Figure 5C. Similarly, the significant decline in *Lactobacilli* from 16.4% in infants to 3.77% in toddlers (Figure 4A) could potentially be attributed to the absence of dairy-only products from the diet of children aged 13–24 months, as illustrated in Figure 5A. This addition of animal-based food with a plant-based diet added a new dimension to the microbial composition in this age group. This emphasizes that, while food type influences composition, age remains a major determining factor in shaping the microbiota.

### Functional outcome of diet and age interplay defining gut microbiota composition

Our in-depth investigation has systematically explored the dynamic shifts in microbial composition influenced by pivotal factors of age and dietary patterns across a diverse spectrum of children spanning ages from birth to 5 years old. We also evaluated the impact of factors such as delivery mode, gender, and type of porridge given, and found no significant differences in microbial composition or diversity based on these variables. Additionally, to analyze whether the socio-economic status of parents affects children’s gut microbial diversity, we partitioned the children into three groups based on their parent’s per month income: low (0–80$), middle (90–200$), and high (210–450$) and microbial diversity was measured within each age group. For the age groups 0–6 and 25–60 months, no significant differences in microbial diversity were found. However, significant variation was observed in the 7–12 months and 13–24 months age groups as detailed in Supplementary Figure S4.

**Figure 6:**
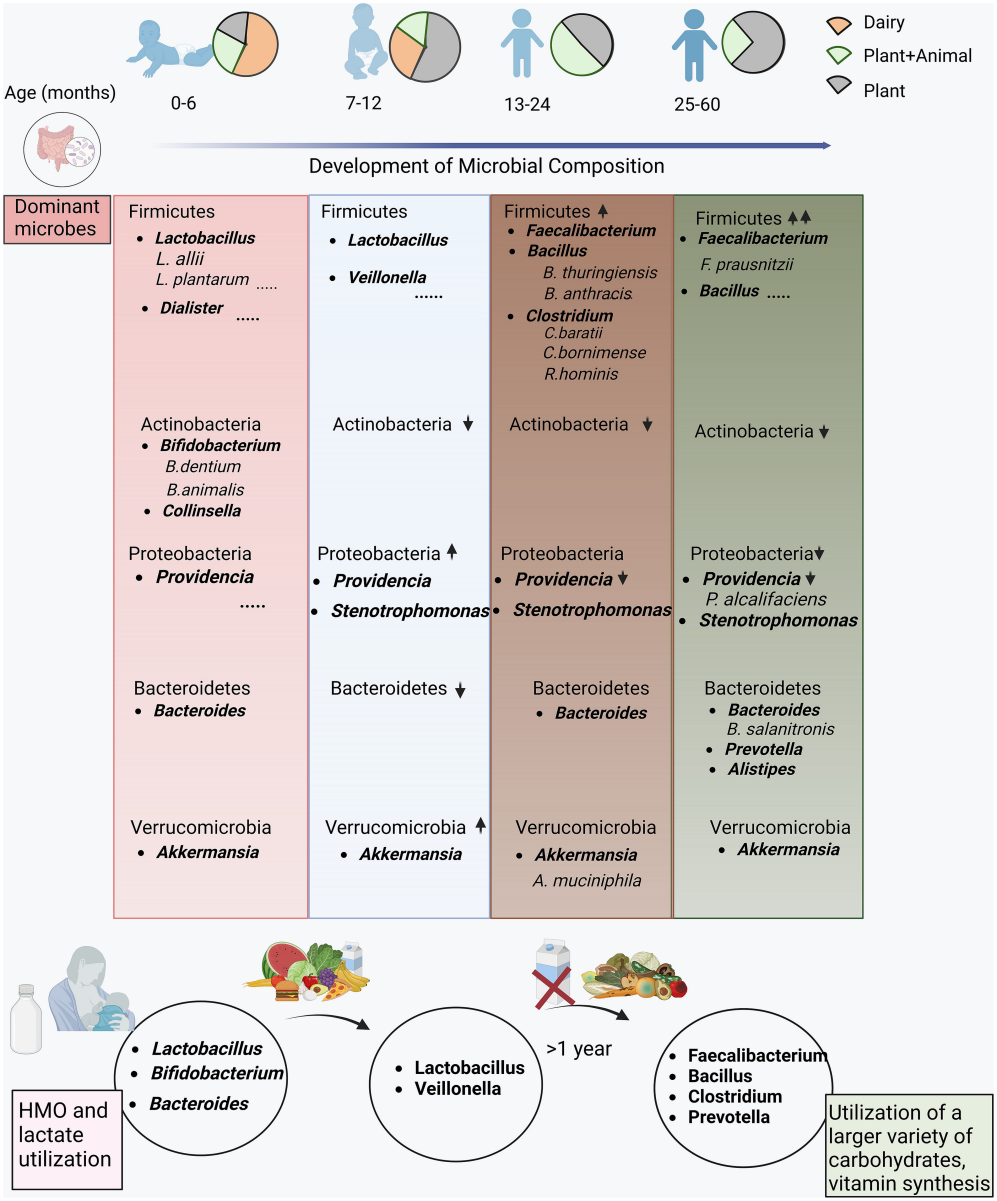
Functional interpretation of the differentially abundant microbes in the study cohort. The encompassing illustration of the evolution of the microbial composition at different hierarchies with the unfolding of dietary habits from early stage (0–6 months) consuming majorly dairy based food transitioning to complementary foods (plant and animal based diet) at the late stage (7–12, 13–24 and 25–60 months).

Figure 6 shows the changes in microbial composition at different levels, highlighting clear patterns. Firmicutes, positioned as the predominant phylum, displayed an escalating abundance with increasing age. However, the specific genera within the phylum Firmicutes underwent nuanced changes across the distinct age groups. Notably, the prevalence of *Lactobacilli* genus and species stood out in infants below 1 year, while *Faecalibacterium*, *Bacillus*, and *Clostridium*, members of the same phylum, and were enriched in the children aged above 1 year. Additionally, the phyla Actinobacteria and Proteobacteria demonstrated a discernible decline with increasing age. Likewise, Bacteroidetes exhibited a consistent enrichment across all the age groups, particularly at early (0–6 months) and later stages (25–60 months old). Within the Bacteroidetes phylum, discernible fluctuations in the abundance of specific genera were evident as children advanced to higher age groups. This was exemplified by a rise in genera such as *Prevotella* and *Alistipes* in the 25–60 month-old children.

A noteworthy observation highlighted the variability in microbiota, particularly during the first year, with the 7–12 age group exhibiting a distinct microbial composition. In the initial 6 months, children in our study predominantly consumed dairy products, resulting in a substantial enrichment of *Lactobacilli* and *Bifidobacteria*. This aligns with the reliance on breast milk and dairy products in the first 6 months, serving as significant sources for *Lactobacilli* and *Bifidobacteria*, prevalent genera in this age group. The transition to solid foods after 6 months fosters a diverse environment for genera and species such as *Faecalibacterium* (*F. prausnitzii*), *Clostridium* (*C. baratii*, *C. bomimens*e), *Bacteroides*, and *Roseburia hominis*, supporting carbohydrate metabolism and vitamin synthesis.

In essence, our study sheds light on the nuanced dynamics of microbial composition, emphasizing the interplay of age and dietary patterns. The findings underscore the intricate shifts in microbial communities during early childhood development, offering valuable insights into the factors shaping the gut microbiota.

## Discussion

Our study investigated the gut microbial composition in children under or equal to the age of 5 years, emphasizing the crucial role of initial gut microbiome colonization in regulating energy extraction and pathogen protection, with potential long-term effects. Notably, our research employs a unique sampling method, encompassing participants aged 0–6, 7–12, 13–24, and 25–60 months, concurrently analyzing their dietary patterns. This metagenomics approach allows us to capture the dynamic nature of gut microbial changes over time in Cameroon an LMIC, offering valuable insights into how age-specific factors and dietary influences shape the microbial landscape in the early years of a child’s life.

Our study aligns with established patterns, showing a consistent trend of increasing microbial alpha diversity with age. Existing research consistently affirms that microbial diversity is lower at birth with the prevalence of aerobic microbes, followed by a gradual increase in diversity favoring anaerobic microbes. This trend is particularly evident from the initial 36 months, with diversity stabilizing by the age of 3 years (Tanaka and Nakayama, 2017). One of our key findings was the variation at the phylum level, with Firmicutes and Bacteroidetes being predominant, while Actinobacteria and Proteobacteria declined with age, particularly in children aged 25–60 months. Researchers observed similar trends in the phyla Firmicutes and Bacteroidetes, as well as Actinobacteria, Proteobacteria, and Verrucomicrobia, in the 18–36 month age group (Tap et al., 2009; Andersson et al., 2008). Previous studies suggest that the increased presence of Firmicutes with age is influenced by the impact of breast milk suppression and the introduction of solid foods. This dietary shift promotes bacterial diversity and the production of short-chain fatty acids, primarily due to Firmicutes’ effective carbohydrate metabolism. Notably, analysis at the genus level within Firmicutes, which includes over 200 genera, reveals enrichment, particularly of *Lactobacilli* during infancy, as documented in existing literature (Rinninella et al., 2019). During infancy, a predominantly dairy-based diet fosters the growth of these microbes, known for their ability to ferment milk oligosaccharides.

On the other hand, the presence of Actinobacteria diminishes after weaning, likely influenced by reduced protein needs and a decrease in genera such as *Bifidobacteria* and *Collinsella* within Actinobacteria, as observed in our data (Yao et al., 2021; Guo et al., 2020). Infants aged 0–6 months typically exhibit higher levels of Bacteroidetes, which could be linked to the gradual rise of *Bacteroides* before weaning. This period sees *Bacteroides* competing with *Bifidobacteria* for dominance in the infant gut. Multiple studies suggest that the elevated presence of Firmicutes and Bacteroidetes signifies the progressive maturation of the gut microbiota with age. However, despite the declining trend with age, infants aged 7–12 months show steep elevation in Proteobacteria abundance, indicating dynamic shifts that warrant further investigation. Additionally, there is a documented gradual transition, characterized by a prevalence of *Clostridium*, *Streptococcus*, *Faecalibacterium*, *Bacillus*, *Ruminococcus*, and *Roseburia* as children progress from infancy to 5 years old. After the introduction of solid foods, the composition of the infant gut microbiota begins to resemble that of adults. This developmental shift over 2–3 years after birth is well-supported by evidence in the literature (Tamburini et al., 2016; Clemente et al., 2012).

Examining deeper at the species level, infants (0–6 months) were identified with *Lactiplantibacillus* (*Lpb. allii* and *Lpb. plantarum*) and *Bifidobacteria* (*Bif. dentium* and *Bif. animalis*), whereas 7–12 month-olds showed higher levels of *Veillonella parvula* and Acinetobacter species (*A. schindleri* and *A. pittii*). *Clostridium* species were notably enriched in children aged 13–24 months, while *Klebsiella*, *Alistipes*, *Faecalibacterium prausnitzii*, and *B. salanitronis* were more abundant in those aged 25–60 months. *F. prausnitzii* is one of the most abundant microorganisms in the intestinal tract of healthy people; it can generate butyrate as an anti-inflammatory to help slow down inflammatory bowel disease (Khan et al., 2012). The maturation of gut microbiota and increasing microbial diversity with age are evident, with studies indicating that introducing complementary foods correlates with heightened bacterial load and diversity.

In our study, we analyzed the impact of different food types (dairy-based, plant-based, and mixed plant and animal) on microbial evolution across various age groups, observing significant variations with age. Thread of findings demonstrate that infants exhibit higher levels of beneficial species, particularly *Bif. breve*, *Bif. bifidum*, and *Bif. longum*, thriving on human milk oligosaccharides (HMOs) which is in line with our data (Bergström et al., 2014; Derrien et al., 2019). This prepares the gut microbiota for lactate and plant-derived glycans metabolism, indicating readiness for simple plant-derived foods (Derrien et al., 2019). *Bifidobacteria* and *Lactobacilli*, acting as probiotics, produce lactic and acetic acids, contributing to protection against pathogenic microbes (Tanaka and Nakayama, 2017; Lew et al., 2013). Consistent findings emphasize the protective role of maternal milk, including immune effectors like immunoglobulin A (IgA), in combating infections in infants (Tanaka and Nakayama, 2017).

Subsequently, children in our study shifted to a primarily plant-based complementary diet after 6 months, we observed shifts in the gut microbiome resembling an adult-like structure. These changes included notable variations in genera across diet groups; for example, the decline of *Lactobacilli* after 6 months was influenced by both age and diet, particularly evident in children with dairy-based diets showing low prevalence. The increased presence of *Klebsiella*, predominantly found in children aged 25–60 months with animal-based diets, highlights the dietary impact on microbial changes as children grow older. A plant-based diet promotes the production of short-chain fatty acids (SCFAs), such as acetate, propionate, and butyrate, through the enrichment of fiber-degrading bacteria like *Prevotella*, *Ruminococcus*, *Faecalibacterium*, and *Eubacterium* (De Filippis et al., 2016; Tomova et al., 2019). Notably, butyrate plays a pivotal role in directly inhibiting intestinal pathogens by reducing oxygen levels in the intestinal epithelium, restricting access to pathogens like *Salmonella typhimurium* (Singh et al., 2022). Furthermore, it stimulates antibacterial activity by promoting the differentiation of monocytes into macrophages, while propionate acts as a safeguard against *Salmonella* invasion by suppressing genes within the *Salmonella* pathogenicity island (Scott et al., 2008; Schulthess et al., 2019; Hung et al., 2013).

Research findings indicate that individuals following a vegetarian diet demonstrate elevated intakes of essential nutrients, including vitamin C, vitamin A, folate, calcium, and magnesium. Gut microorganisms, facilitated by retinol dehydrogenase 7 (Rdh7), convert dietary vitamin A into bioactive retinoic acid. Studies with mice lacking Rdh7 reveal that reduced retinoic acid levels are associated with diminished IL-22, a critical signal in the intestinal antibiotic response (Xiao et al., 2022). Veldhoen and Brucklacher-Waldert (2012) demonstrate that retinoic acid derived from dietary vitamin A, produced by CD103^+^ dendritic cells and intestinal epithelial cells (IECs), significantly influences the differentiation of T cells and B cells (Xiao et al., 2022). Vitamin A is essential for immune function, impacting the differentiation and proliferation of the immune cells. Moreover, plant-based fats appear to positively influence the abundance of beneficial bacteria, including *Bifidobacteria*, *Roseburia*, and *Faecilibacterium*, contributing to overall health benefits (Kim and Choi, 2021). This is summarized in Table 1. This shift is associated with higher bacterial load and diversity, elevated total short-chain fatty acid levels, and a dominance of Bacteroides/Firmicutes, adept at breaking down complex carbohydrates which have been deciphered through various studies.

**Table 1:** List detailing bacterial genera and species along with their respective biological functions for different age groups, categorized as under and over 6 months.

Age (months)	Significant species	Genus	Basic features	Biological function
Prevalent in <6 months	*Bif. dentium*, *Bif. animalis*	*Bifidobacteria*	Gram-positive, facultative anaerobic	Produce GABA, secondary metabolite, and IgA, used as probiotic, anti-inflammatory effects, improve gut mucosal barrier
	*Lpb. oligofermentas*, *Lacticaseibacillus buchneri*, *Lpb. allii* and *Lpb. plantarum*	*Lactobacilli*	Gram-positive, anaerobic	Produce GABA, lactic acid and secondary metabolite, used as probiotic
		*Bacteroides*	Gram-negative, strict anaerobic	Activate CD4^+^ T cells
Prevalent in >6 months	*V. parvula*	*Veillonella*	Gram-negative, anaerobic	Ability to produce SCFA like propionate, acetate, and CO_2_ as the major end products of glucose, lactate, and glycerol fermentation
	*C. baratii*, *C. botulinum*, *C. bornimenes*, *C. beijerinckii*	*Clostridium*	Gram-positive, strict anaerobic	Butyrate-producing taxa, promote generation of Th17 cells
	*F. prausnitzii*	*Faecalibacterium*	Gram-positive, anaerobic	Potential anti-inflammatory and SCFA producing bacteria
	*R. hominis*	*Roseburia*	Gram-positive, anaerobic	SCFA producing bacteria
	*K. pneumoniae*	*Klebsiella*	Gram-negative, facultative anaerobic	Mainly found in meat rich diet, and capable of fermenting lactose, found to be associated with children’s infections like pneumonia, bloodstream infection, wound or surgical site infection
	*B. thuringiensis*, *B. anthracis*	*Bacillus*	Gram-positive, anaerobic	
	*A. hallii*	*Anaerobutyricum*	Gram-positive, anaerobic	Can convert a potentially damaging acid (e.g., lactic acid) into butyrate, a short-chain fatty acid (SCFA), which has known beneficial effects on glucose metabolism
	*P. jejuni*	*Prevotella*	Gram-negative	Potential anti-inflammatory bacteria, mostly associated with high fiber diet through fermentation
	*A. muciniphila*	*Akkermansia*	Gram-negative, strictly anaerobic	Mucin-degrading, present abundantly in the guts of healthy adults, an important marker for gut health; a commensal microorganism, anti-inflammatory effects

A high-fiber diet is associated with *Prevotella* abundance, crucial for early-life microbiota development, and adapting to dynamic intestinal conditions. Our study reveals a complex interplay between age and diet in shaping gut microbial composition. Examining microbial variability in relation to socioeconomic factors while controlling for age and diet, we found that parental economic status does not significantly influence microbial composition during early infancy and after the microbiota has matured beyond 2 years. The significant variation in the diversity of the gut microbiota within the group of children aged 7–12 months observed in this study could be related to the introduction of complementary foods into their diet. Indeed, above 7 months, the majority of mothers declared to supplement breast milk with complement foods including traditional homemade porridge or/and commercialized porridge. This newly introduced diet within a specific microbiota and ingredients may contribute to modify the gut microbiota of these children. A similar observation was made by Di Profio et al. (2022). The authors highlighted that introduction of novel food in children diet modified their gut microbiota. Likewise, children in the age group of 13–24 months are in the nutritional transition stage characterized by introduction of the solid household diet like rice, fufu, potatoes in addition to complementary diet sometime made of porridge, milk and others. This variation in the children diet also contribute to significant modification of the gut microbiota diversity. Meanwhile, within the age group 25–60 of months, we noticed that the complementary diet was subtracted from the children diet. They rely only on the household meals. This modification in the diet through subtraction of foods that might diversify the gut microbiota like milk, porridge, fruit (compote) could justify the weak impact on the diversity of gut microbiota observed in the age group 25–60 of months (Di Profio et al., 2022). However, some differences in microbial dynamics were observed in the 7–12 months and 13–24 months age groups, indicating that socioeconomic factors may have a modest impact on microbial composition during these transitional periods.

We would like to mention that the study was not focused on a specific population in Cameroon. The city of Yaoundé is the capital of Cameroon and is a cosmopolitan city with the population originating from the 10 regions of the country. During the enrolment of participants, the variable region was not an inclusion criterion, participants originated from the 10 regions of the country. Besides, the 7 district hospitals in Yaoundé are free of charge and accessible for every population independent of their economic status. This study therefore gives an overview of what could be expected in the country. Furthermore, the classification we gave regarding dietary patterns is not particular to Cameroon, but also applies to many other urbanized cities or countries in Africa and in other parts of the world. Our study highlights that incorporating dietary fibers into children’s nutrition can promote the growth of fiber-degrading bacteria such as Prevotella, Ruminococcus, Faecalibacterium, and Eubacterium. These bacteria are known producers of short-chain fatty acids (SCFAs) like acetate, propionate, and butyrate, which provide numerous health benefits, including anti-inflammatory, immunoregulatory, anti-obesity, anti-diabetes, anticancer, cardiovascular protective, hepatoprotective, and neuroprotective effects. Building on these findings, future research should explore the long-term effects of microbiota-targeted nutritional interventions in LMICs. Building on these findings, future research should explore the long-term effects of microbiota-targeted nutritional interventions in LMICs. Longitudinal studies are essential to evaluate how dietary modifications and fortified foods influence gut microbiota development and malnutrition recovery over time.

Addressing malnutrition in Cameroon and similar low- and middle-income countries requires a comprehensive approach that integrates nutritional interventions with an understanding of gut microbiota development. In collaboration with UNICEF, the Cameroonian government has launched several nutrition programs aimed at reducing malnutrition. These include community-based food fortification, distribution of ready-to-use therapeutic foods, micronutrient supplementation (e.g., zinc, iron, iodine, and vitamin A), deworming programs, complementary food enrichment, and the promotion of exclusive breastfeeding for infants aged 0–6 months. These initiatives aim to tackle key challenges such as food insecurity, poor feeding practices, high rates of childhood diseases, and limited access to clean water, sanitation, and healthcare (Sobngwi-Tambekou et al., 2024).

Despite the extensive reach of these programs, they do not yet account for the role of gut microbiota in nutrient absorption. The findings of this study underscore the importance of early-life gut microbiota in regulating energy extraction, nutrient assimilation, and pathogen defense. Current programs could be enhanced by incorporating microbiota-targeted strategies. For instance, the addition of prebiotic fibers or probiotic strains to fortified foods may help optimize gut microbiota composition, thereby improving nutrient absorption and immune resilience. Community-based initiatives should also focus on promoting diverse and nutrient-rich diets through locally available foods. Education campaigns targeting caregivers on proper complementary feeding practices, hygiene, and sanitation could further improve gut health outcomes. Additionally, integrating microbiota-based diagnostics into nutritional assessments may help identify at-risk populations and tailor interventions accordingly.

In addition, other factors such as delivery mode, geography, environmental factors, genetics, and maternal health may also influence microbial composition. These factors present potential limitations to our study and should be considered in the future research in this direction. Conversely, our study utilized a DNA-based approach to identify microbial composition, specifically employing the 16S rDNA gene library to provide a comprehensive overview of the microbial community. However, this method does not differentiate between live and dead bacterial species. Future studies could benefit from combining DNA and RNA analyses, such as RNA-seq, to offer a more holistic view of both microbial diversity and the active components within these communities. This combined approach would enhance our understanding of the functional roles of the microbiota.

## Conclusion

As children grow, their gut microbiota develops in a manner similar to adults, with diet playing a crucial role in shaping specific microbial profiles. In early infancy (0–6 months), consumption of dairy products, including breast milk or formula, enriches the *Bifidobacteria* and *Lactobacilli* genera. The transition from breastfeeding to solid foods increases microbial diversity and shifts the microbiota towards an adult-like composition, dominated by genera such as *Clostridium*, *Acinetobacter*, *Faecalibacterium*, *Streptococcus*, *Bacillus*, *Roseburia*, and *Klebsiella*. Subsequently, most children transitioned to a primarily plant-based complementary diet after 6 months. A plant-based diet promotes the production of short-chain fatty acids (SCFAs), such as acetate, propionate, and butyrate, through the enrichment of fiber-degrading bacteria like *Prevotella*, *Ruminococcus*, *Faecalibacterium*, and *Eubacterium*. Moreover, plant-based fats appear to positively influence the abundance of beneficial bacteria, including *Bifidobacteria*, *Roseburia*, and *Faecalibacterium*, contributing to overall health benefits. The increased presence of *Klebsiella*, predominantly found in children aged 25–60 months with animal-based diets, highlights the dietary impact on microbial changes as children grow older. Our study contributes to the growing body of literature linking the dual influence of age and diet on the gut microbiota’s transition from infancy to childhood, highlighting the need for a comprehensive approach to understanding and promoting optimal gut health in pediatric populations. Further research involving larger cohorts and incorporating RNA sequencing to investigate transcriptionally active microbes will help elucidate the mechanisms by which the functional microbiome develops in children, particularly in low- and middle-income countries like Cameroon.

## Data Availability

The data presented in the study are deposited in the NCBI SRA repository, accession number PRJNA1048169 (https://www.ncbi.nlm.nih.gov/sra/PRJNA1048169).
