# Supplementary material for: Exploring the influence of age and diet on gut microbiota development in children during the first 5 years: a study from Yaoundé, Cameroon
Source: Front Microbiol. 2024 Dec 18;15:1512111. doi: 10.3389/fmicb.2024.1512111 (PMC11688346; doi:10.3389/fmicb.2024.1512111)
Supplement: Supplementary file 1 [file Supplementary_file_1.zip › Supplementary Figures 1-4.DOCX]

Supplementary Material

**Exploring the Influence of Age and Diet on Gut Microbiota Development in Children during the First 5 Years, with a Focus on Low- and Middle-Income Countries (LMICs)**

Jorelle Jeanne B. Adjele^4,5†^ Priti Devi^1,2,†^, Pallawi Kumari^1,3^, Aanchal Yadav^1,2,^, Alex D. Tchuenchieu Kamgain^5,6^, Hippolyte T. Mouafo^5^, Gabriel N. Medoua^5^, Justin J. N. Essia^4^, Nar Singh Chauhan^7^, Rajesh Pandey^1,2,^*

*Corresponding author

**Supplementary Figures**
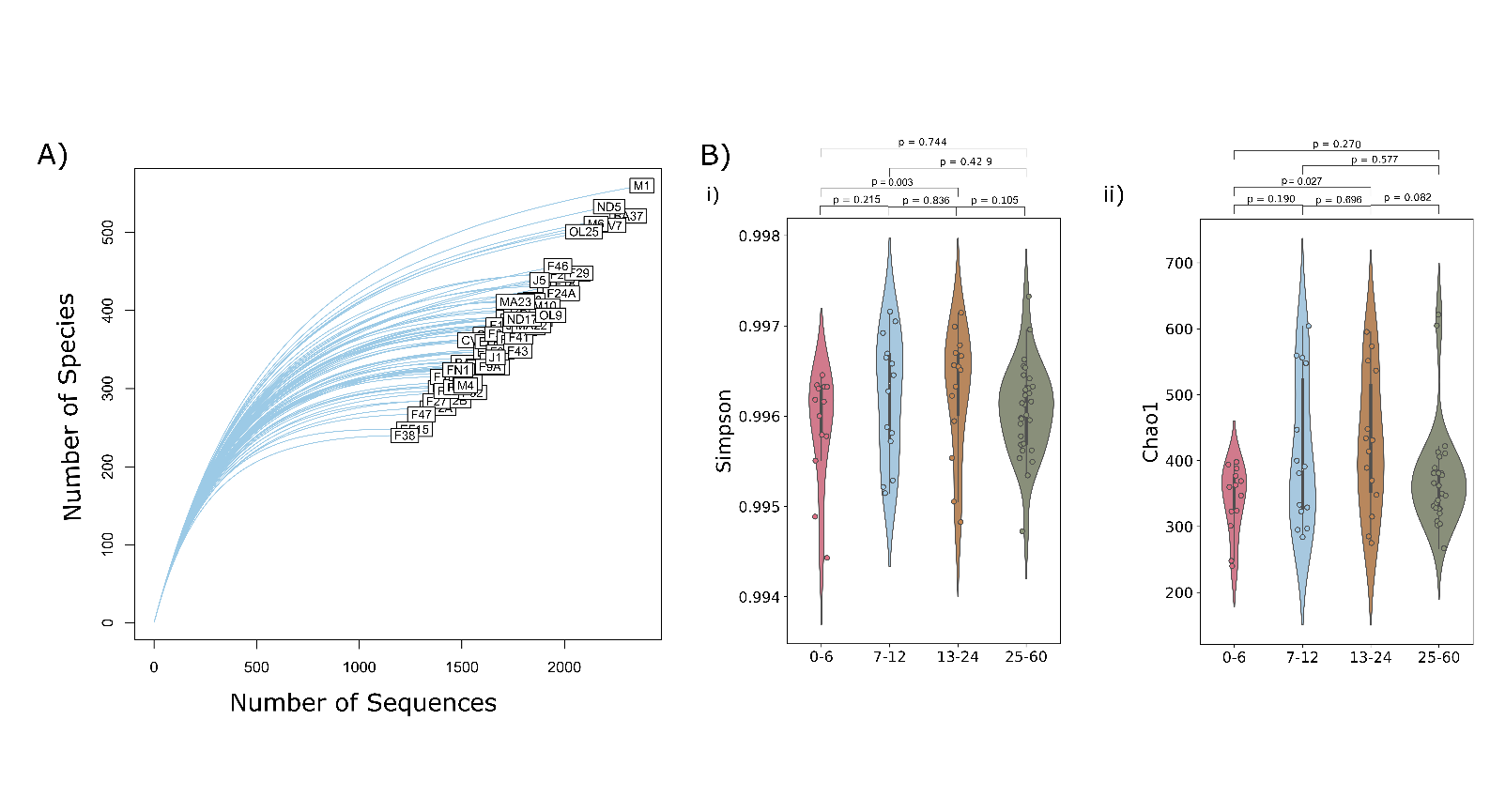


**Supplementary Figure 1: Compendium of the diversity analysis: (A)** Average of sequences of individual samples. **(B)** Alpha diversity from 0 to 60 months of infants’ age was measured (i) Simpson, (ii) Chao1

**
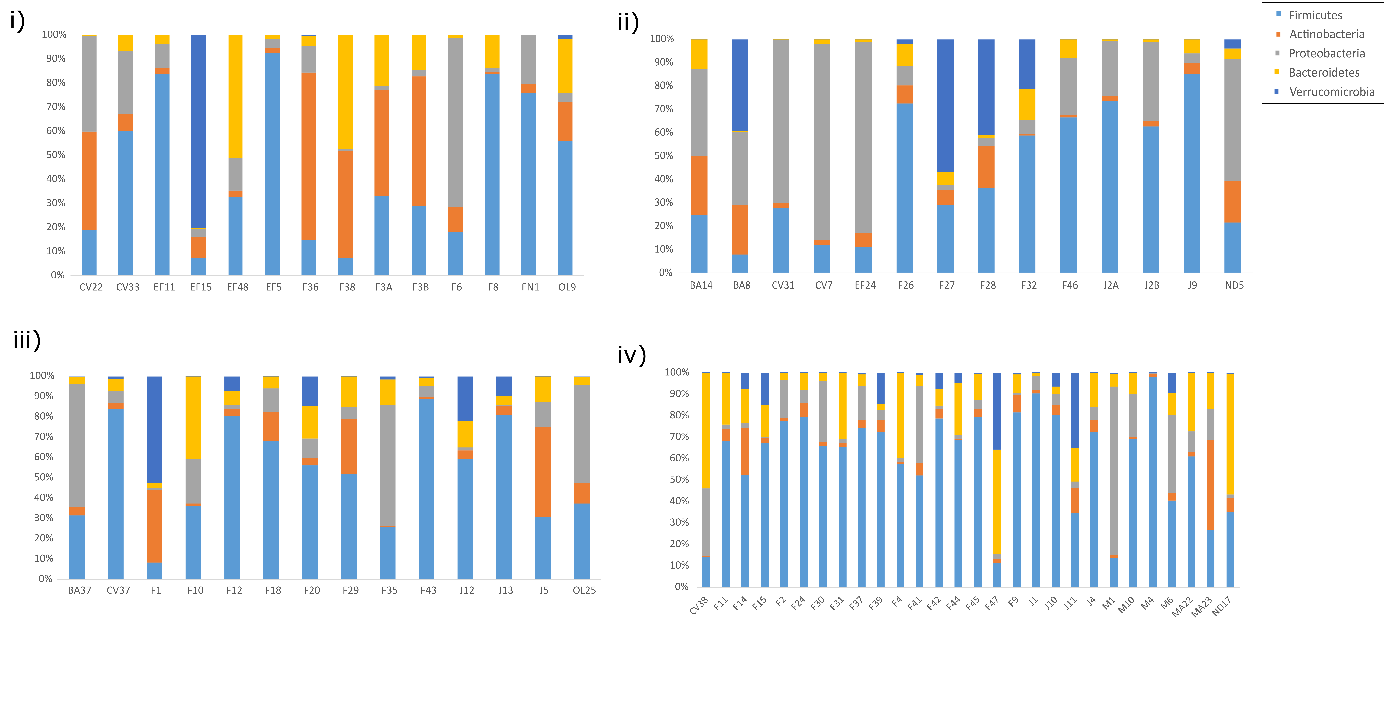
**

**Supplementary Figure 2: Illustration of Microbial Profiles at Phyla levels per sample across various age groups (0-6, 7-12, 13-24, and 25-60).** (i) The relative abundances of the 5 major phyla in 0-6 age group (ii) 7-12 (iii) 13-24 (iv) 25-60 month age groups

**
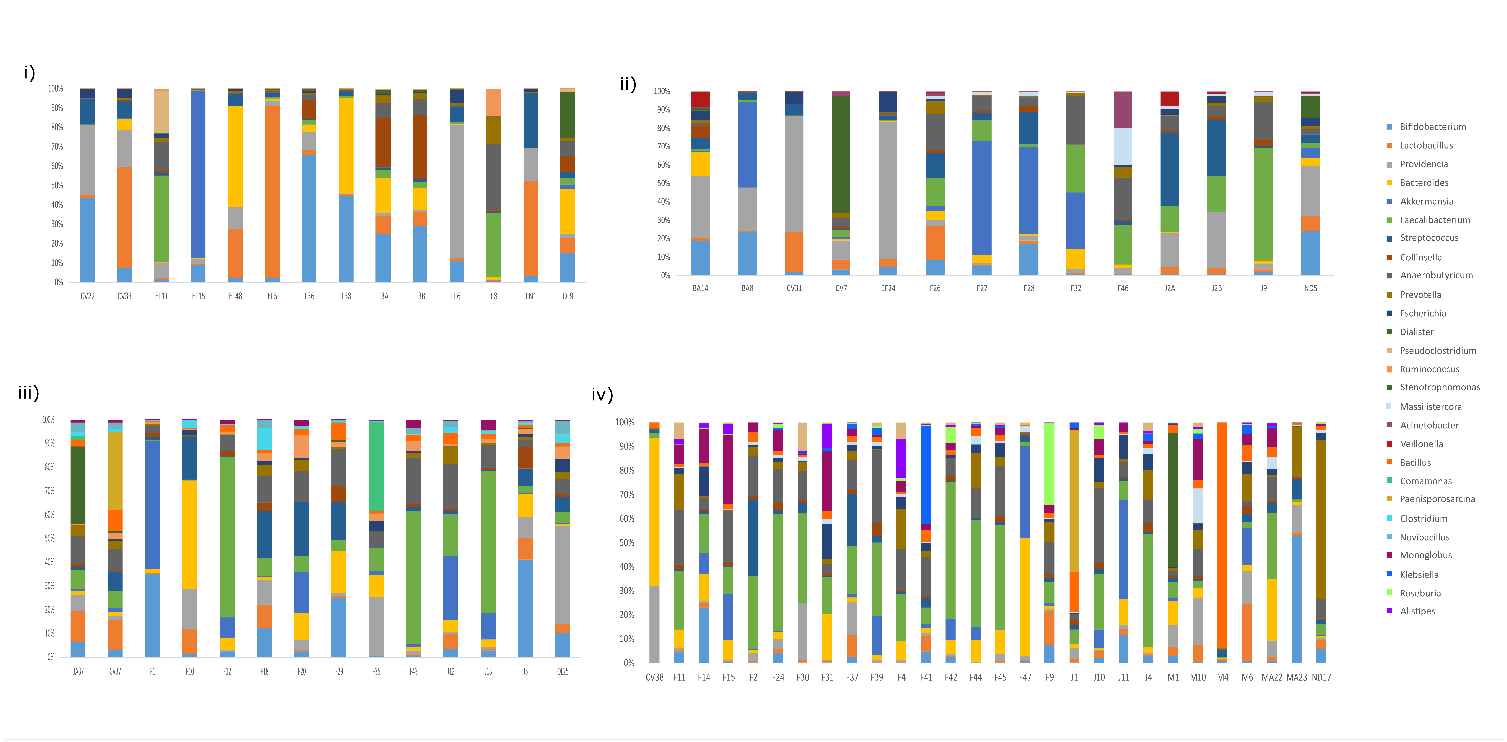
**

**Supplementary Figure 3**: **Illustration of Microbial Profiles at Genera Taxonomic levels across various Age groups (0-6, 7-12, 13-24, and 25-60) per sample.** (i) The relative abundances of the 5 major Phyla in 0-6 month (ii) 7-12 (iii) 13-24 (iv) 25-60 month age groups


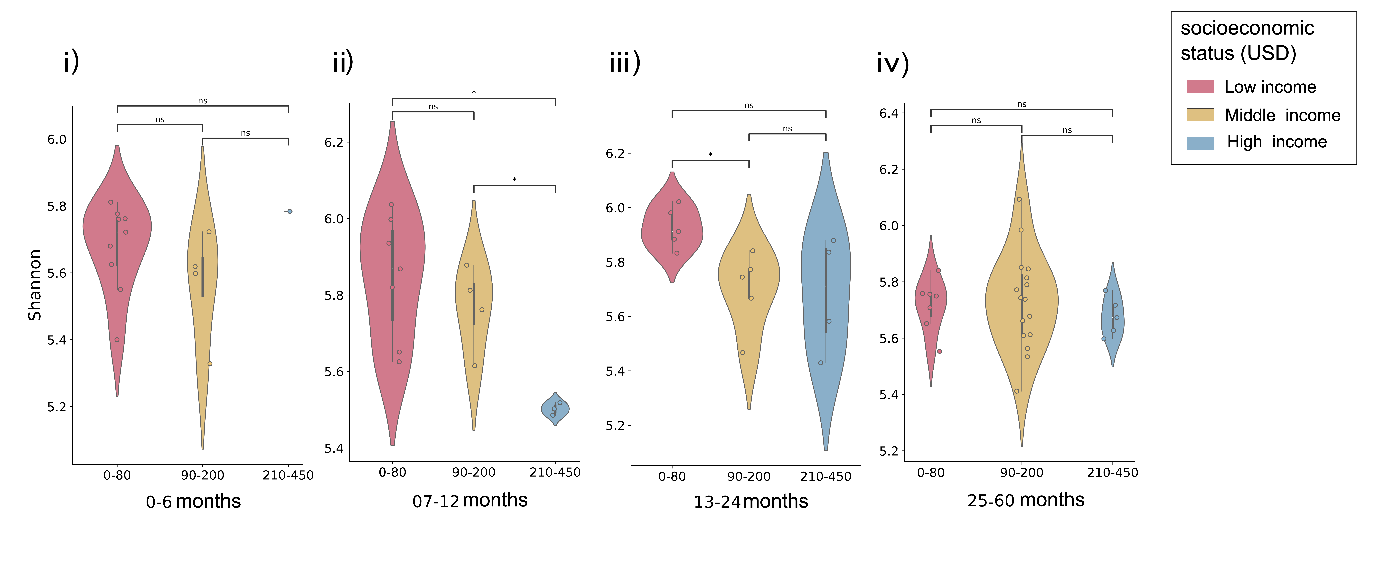


**Supplementary Figure 4: Illustration of Shannon alpha microbial diversity based on socioeconomic status in each age group:** (i) 0-6 months, (ii) 7-12 months, (iii) 13-24 months, and (iv) 25-60 months
